# Case Report: aleukemic mast cell leukemia with *KIT* p.V559G mutation, CD25-negative immunophenotype, and complex karyotype

**DOI:** 10.3389/fonc.2026.1878036

**Published:** 2026-07-08

**Authors:** Longyi Zhang, Lijing Jiang, Xiaowei Zhang, Yan Lu, Xiaoxia Wang

**Affiliations:** 1 Clinical Laboratory, Affiliated Dongyang Hospital of Wenzhou Medical University, Dongyang, Zhejiang, China; 2 Department of Pathology, Affiliated Dongyang Hospital of Wenzhou Medical University, Dongyang, Zhejiang, China; 3 Department of Hematology, Affiliated Dongyang Hospital of Wenzhou Medical University, Dongyang, Zhejiang, China

**Keywords:** aleukemic mast cell leukemia, case report, CD25, *KIT* mutation, mast cell leukemia

## Abstract

Mast cell leukemia (MCL), the most aggressive and often fatal subtype of systemic mastocytosis, is predominantly driven by *KIT* mutations, with the *KIT* p.D816V mutation being the most prevalent. Rare noncanonical *KIT* mutations remain poorly characterized, particularly when combined with a CD25−negative immunophenotype and high−risk cytogenetics. Through this case report, we aimed to enrich the molecular spectrum of MCL, emphasize the value of integrated diagnosis, and provide a reference for individualized treatment of older patients with high-risk MCL. This case involves an 81−year−old woman, with aleukemic MCL, who presented with dizziness and back pain. Severe pancytopenia was present, with 3% of atypical mast cells in the peripheral blood and 69.5% of abnormal mast cells in the bone marrow. Flow cytometry revealed a unique CD117^bri^, CD9^bri^, CD203c^+^, CD2^+^, CD25^-^, and HLA−DR^-^ immunophenotypes. Karyotyping revealed complex clonal abnormalities, including monosomy 7. Next−generation sequencing identified a rare activating *KIT* p.V559G mutation (variant allele frequency, 42%), which is uncommon in MCL. Despite advanced age, life−threatening complications (sepsis, gastrointestinal bleeding, and myocardial infarction), and dismal prognostic features, the patient achieved clinical improvement with partial hematologic recovery on low−dose imatinib plus intensive supportive care and remained stable without evidence of disease progression during a four month follow-up period. This case represents a rare instance of aleukemic MCL cases with a *KIT* p.V559G mutation, CD25 negativity, and a complex karyotype, underscoring the importance of a comprehensive diagnostic workup and expands the clinical and molecular spectrum of MCL. Individualized low−intensity targeted therapy may offer short−term disease control in older patients with high−risk MCL, though long−term survival benefit remains unproven.

## 1 Introduction

Systemic mastocytosis (SM) is a group of clonal hematological neoplasms. Mast cell leukemia (MCL) is the most aggressive and fatal subtype of SM, with an extremely poor prognosis (1, 2). The canonical *KIT* p.D816V mutation is the dominant driver in most SM/MCL cases, while alternative *KIT* mutations are relatively rare and less well characterized (3). Aberrant CD2/CD25 expression is a key immunophenotypic feature of neoplastic mast cells; however, CD25-negative MCL is rare and prone to misdiagnosis (4). Complex karyotype and monosomy 7 further worsen the prognosis (5). Further, rare noncanonical *KIT* mutations remain poorly characterized, particularly when combined with a CD25−negative immunophenotype and high−risk cytogenetics.

Here, we report an extremely rare case of aleukemic MCL in an older patient with *KIT* p.V559G mutation, CD25-negative immunophenotype and complex cytogenetics. This case aimed to enrich the molecular spectrum of MCL, emphasize the value of integrated diagnosis, and provide a reference for individualized treatment of older patients with high-risk MCL.

## 2 Case description

### 2.1 Clinical data

An 81-year-old female was admitted to our hospital in December 2025, presented with dizziness and back pain that had persisted for one week. The patient initially received a flurbiprofen gel patch for back pain management at another hospital. However, no clinical relief was achieved, prompting the patient to seek further medical evaluation at our institution. The patient’s medical history was notable for hypertension. Physical examination revealed pallor of the skin and mucous membranes, but no rash, hepatosplenomegaly, or lymphadenopathy was identified on palpation. Chest computed tomography (CT) revealed mild pleural effusion. Abdominal ultrasound of the liver, gallbladder, spleen and pancreas showed no obvious abnormalities.

### 2.2 Laboratory examinations

**Figure 1:**
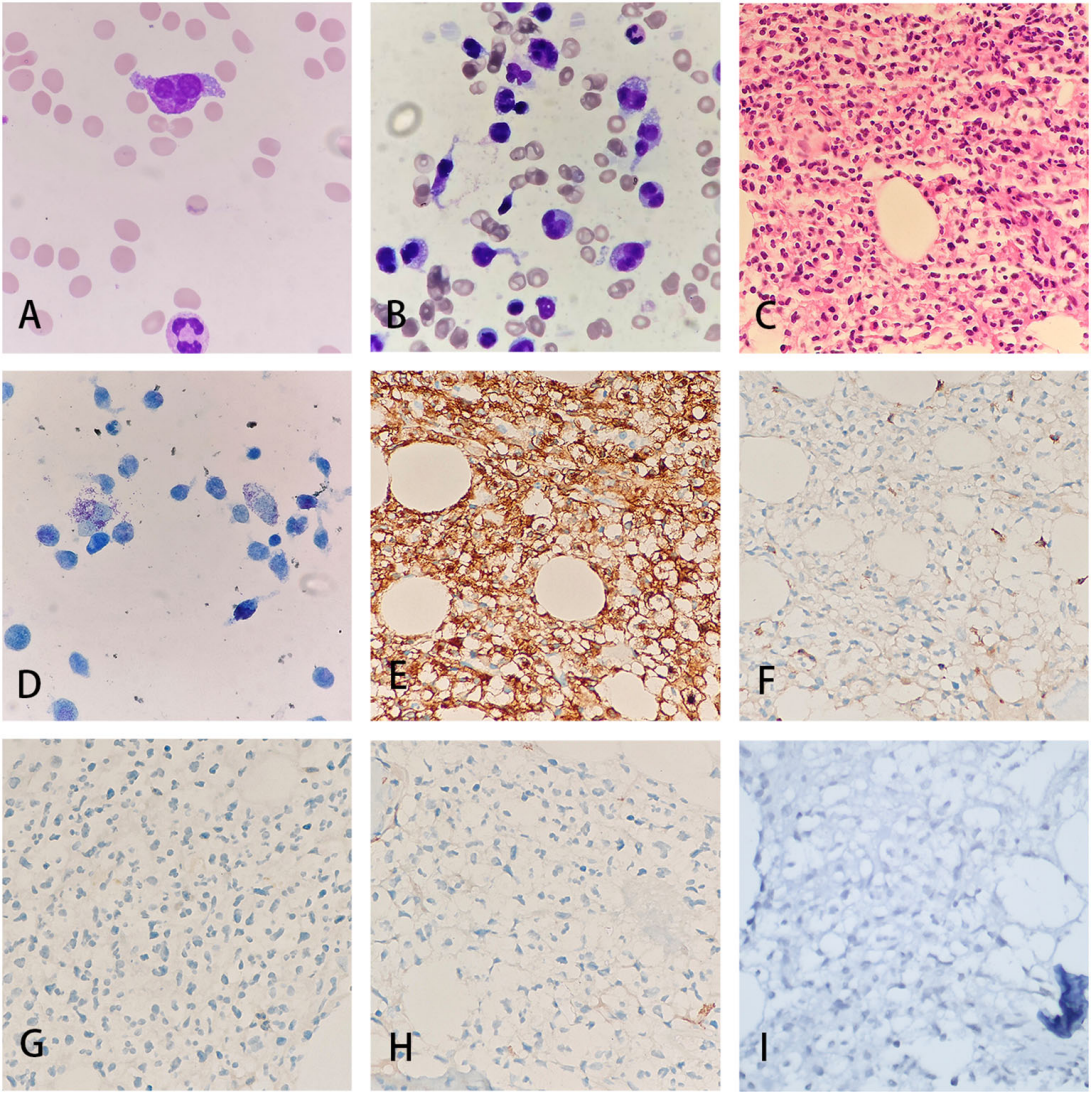
Morphological and pathologic findings of the bone marrow and peripheral blood. **(A)** Peripheral blood smear showing atypical bilobed cells suspicious for mast cells (Wright–Giemsa stain, 1000×). **(B)** Bone marrow aspirate smear showing clusters of atypical mast cells with bilobed or multilobed nuclei, abundant cytoplasm containing purple-red granules, and vacuoles (Wright–Giemsa stain, 1000×). **(C)** Bone marrow biopsy demonstrating diffuse and dense infiltration of abnormal cells replacing the normal hematopoietic tissue (hematoxylin and eosin stain, 200×). **(D)** Toluidine blue staining of the bone marrow showing metachromatic staining of mast cell granules (1000×). **(E–I)** Immunohistochemical staining of the bone marrow revealing strong positivity for CD117 **(E)** and focal positivity for CD2 **(F)**, negative for CD30 **(G)**, CD34 **(H)**, and tryptase **(I)** (400×).

Complete blood count revealed a white blood cell count of 17.49×10^9^/L, with a differential count of 72% neutrophils, 20% lymphocytes, 5% monocytes, and 3% atypical bilobed cells suspicious for mast cells (**Figure 1A**). Additional complete blood count findings included a hemoglobin level of 42 g/L and a platelet count of 23×10^9^/L. Coagulation profiling revealed a prolonged prothrombin time (23.9 seconds), prolonged thrombin time (24.5 seconds), and an elevated D-dimer level (18.28 μg/mL). Fasting blood glucose level was moderately elevated at 8.41 mmol/L. Renal function (assessed by uric acid, urea, and creatinine levels) and liver function (assessed by alanine aminotransferase, aspartate aminotransferase, gamma-glutamyl transferase, alkaline phosphatase, and total bilirubin levels) were within normal reference ranges. Lactate dehydrogenase was elevated at 336 U/L. Serum tryptase levels were not assayed due to limitations in laboratory capabilities.

### 2.3 Bone marrow examinations

**Figure 2:**
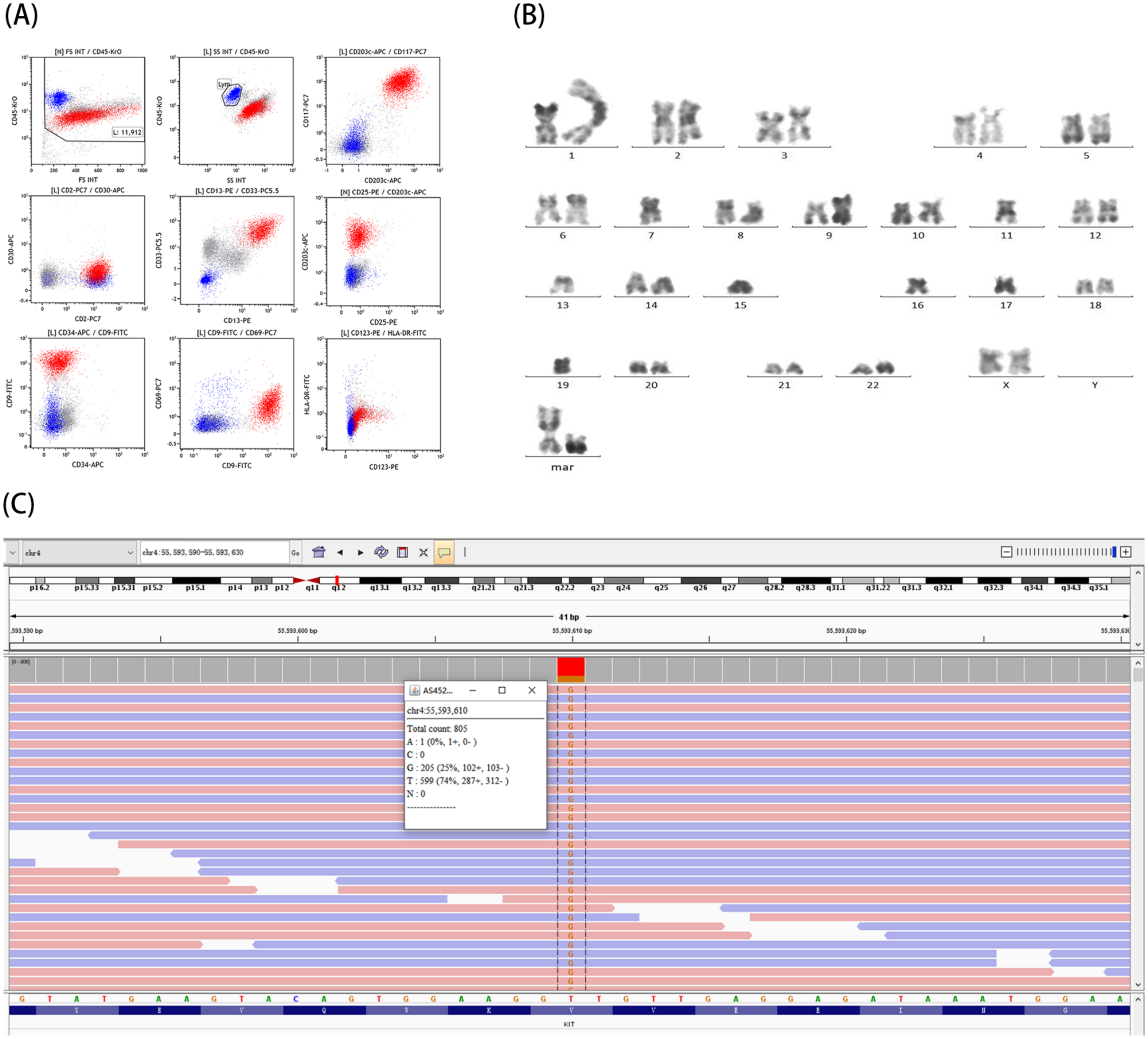
Immunophenotypic, cytogenetic, and molecular genetic characterizations. **(A)** Flow cytometric analysis of bone marrow. The abnormal mast cell population (highlighted in red) shows high forward and side scatter profiles and an immunophenotype of CD117^bri^, CD203c^+^, CD2^+^, CD13^+^, CD33^+^, CD25^-^, HLA-DR^-^, and CD123^-^. **(B)** Representative karyogram revealing a complex karyotype of 46, XX, der (1), -7, add(9p), -11, -13, -15, -16, -17, -19, +mar1, +mar2, inc[20]. **(C)** Next-generation sequencing electropherogram demonstrating the heterozygous mutation c.1676T>G (p.V559G) in exon 11 of *KIT*.

A bone marrow aspirate smear revealed hypercellular, nucleated bone marrow cells. A predominant population (69.5%) of abnormal cells was observed, which were distributed in scattered, clustered, or aggregated patterns. These cells exhibited a round to ovoid morphology with irregular nuclei. Bilobed, multinucleated, and lobulated nuclei were frequently observed, with some demonstrating twisting, folding, deformity, and indistinct nucleoli. The cytoplasm was moderate to abundant, with pseudopodal projections, a greyish-blue hue, and contained varying numbers of unevenly sized purple-red granules and vacuoles (**Figure 1B**). Bone marrow biopsy revealed diffuse and dense infiltration of abnormal cells within the bone marrow cavity, which replaced the normal hematopoietic parenchyma (**Figure 1C**). Bone marrow toluidine blue staining demonstrated focal positive staining in the abnormal cell population (**Figure 1D**). Immunohistochemical staining revealed strong immunoreactivity for CD117 (encoded by *KIT*), focal positivity for CD2 in scattered individual cells, and negative staining for CD30, CD34, and tryptase (**Figures 1E–I**). Flow cytometry demonstrated that abnormal cells constituted 45.45% of the total bone marrow nucleated cells, displaying high forward and side scatter profiles. The immunophenotypic profile was characterized by CD117^bri^, CD203c^+^, CD2^+^, CD30^-^, CD13^+^, CD33^+^, CD25^-^, CD9^bri^, CD34^-^, CD69^dim^, HLA-DR^-^, and CD123^-^ expression, which was consistent with the phenotypic features of abnormal mast cells (**Figure 2A**).

### 2.4 Cytogenetic and molecular biological examinations

Cytogenetic analysis revealed a karyotype of 46, XX, der(1), -7, add(9p), -11, -13, -15, -16, -17, -19, +mar1, +mar2, inc[20], which was indicative of complex clonal chromosomal abnormalities (**Figure 2B**). Next-generation sequencing revealed a missense mutation, c.1676T>G (p.V559G), located in exon 11 of *KIT*, was detected with a variant allele frequency of 42% (**Figure 2C**). No other common concomitant mutations were identified, such as *SRSF2*, *ASXL1*, *RUNX1*, *TET2*, or *NPM1*.

### 2.5 Diagnosis

Diagnosis was established in accordance with the World Health Organization 2022 diagnostic criteria (6).

#### 2.5.1 Diagnosis of SM

The patient met the diffuse and dense mast cell infiltration on bone marrow biopsy, a major criterion. They also met four minor criteria: >25% of mast cells in the bone marrow aspirate exhibited atypical morphology, abnormal mast cells expressed CD2, and detection of an activating *KIT* gene mutation.

#### 2.5.2 Subclassification of MCL

All the following criteria were satisfied: confirmation of SM as outlined above, diffuse and dense mast cell infiltration on bone marrow biopsy, proportion of abnormal (immature) mast cells in the bone marrow aspirate ≥20% (69.5% in the present case), and proportion of abnormal mast cells in the peripheral blood smear <10% (3% in the present case), consistent with the diagnosis of “aleukemic” MCL. Notably, according to the International Consensus Classification (ICC) 2022 (7), the distinction between leukemic and aleukemic forms based purely on the percentage of circulating mast cells is considered of uncertain clinical relevance, as both subtypes carry an equally poor prognosis.

The patient also presented with severe anemia and thrombocytopenia (defined as “C findings”) as well as a high-risk complex karyotype.

### 2.6 Treatment and follow-up

The patient was admitted to our hospital on December 14, 2025. During hospitalization, the patient’s clinical condition was critical with the sequential development of multiple organ complications and acute events. On December 24, 2025, she developed persistent hyperthermia that rapidly progressed to severe pneumonia, sepsis, and type II respiratory failure. Broad-spectrum anti-infective therapy combined with anti-inflammatory treatment was promptly initiated, including meropenem (1 g intravenously every 6 h) between December 24, 2025 and January 5, 2026 for antibacterial coverage, and caspofungin (70 mg loading dose on day 1, followed by 50 mg intravenously once daily) was administered between December 25, 2025 and January 5, 2026 for antifungal prophylaxis and treatment. Concurrently, methylprednisolone (40 mg every 12 h) was administered to suppress the excessive inflammatory response between December 24 and 28, which was reduced to 40 mg once daily between December 29, 2025 and January 1, 2026 following clinical stabilization, and subsequently tapered gradually. After discontinuing caspofungin on January 5, 2026, fluconazole was substituted for the long-term prophylactic antifungal therapy.

Following a definitive diagnosis of MCL, targeted therapy was initiated on December 26, 2025, with imatinib 100 mg​ administered orally once daily. The dose was maintained at 100 mg daily throughout the treatment course and was not escalated. Severe treatment- and disease-related complications occurred sequentially during the course of targeted therapy. On December 28, the patient developed upper gastrointestinal bleeding, as evidenced by positive occult blood in both vomitus and stool. Endoscopy was deferred due to critical illness and high procedural risk. Based on the medication timeline, clinical presentation, and laboratory findings, the bleeding was most likely attributable to imatinib-induced mucosal injury compounded by disease-related coagulopathy. Empirical management with fasting, parenteral nutrition, somatostatin, and etamsylate achieved gradual hemostasis. Acute myocardial infarction was diagnosed on December 31, and atorvastatin calcium was administered for lipid modulation and plaque stabilization in conjunction with hydrotalcite for gastric mucosal protection. After complete stabilization of the upper gastrointestinal bleeding, clopidogrel bisulfate (75 mg) was administered orally on January 7, 2026, as antiplatelet therapy. Detailed timelines of the clinical events, complications, and corresponding management are summarized in **Table 1**.

**Table 1:** Timelines of clinical events, complications, and comprehensive management.

Dates (2025–2026)	Key clinical events & complications	Antimicrobial & anti-infective management	Targeted & anti-inflammatory therapy	Supportive & complication-specific management
December 14	Hospital admission for dizziness and back pain			Initial evaluation and supportive care
December 24	Onset of hyperthermia, progressing to severe pneumonia, sepsis, and type II respiratory failure	Meropenem (1 g IV q6h) initiated. Methylprednisolone (40 mg IV q12h) initiated.		Intensive respiratory and circulatory support
December 25		Caspofungin added (70 mg load, then 50 mg IV qd)		
December 26	Definitive diagnosis of MCL (*KIT* p.V559G+) established		Imatinib (100 mg PO qd) initiated	
December 28	Onset of upper gastrointestinal bleeding		Methylprednisolone dose reduced (to 40 mg IV qd from Dec 29)	Gastrointestinal bleeding management: Fasting, parenteral nutrition, somatostatin, etamsylate
December 31	Diagnosis of acute myocardial infarction			Myocardial infarction management: Atorvastatin, hydrotalcite
January 1	Clinical condition stabilized	Methylprednisolone was tapered off.		
January 5		Caspofungin stopped. Fluconazole started for prophylaxis		
January 7	Gastrointestinal bleeding controlled			Antiplatelet therapy: Clopidogrel (75 mg PO qd) added
January 13	Bone marrow re-assessment: Morphologic remission; MRD: ten abnormal cells/47,875 nucleated cells			
January 14	Clinical recovery, discharged from the hospital			Outpatient follow-up planned
January 14 – May 28	Outpatient follow-up (four months): Condition stable, no progression		Imatinib continued	Regular monitoring

IV, intravenous; MCL, mast cell leukemia; MRD, minimal residual disease; PO, orally; q6h, every 6 h; q12h, every 12; qd, once daily.

After comprehensive treatment, the patient’s clinical symptoms improved substantially. Bone marrow re-examination and multiparametric flow cytometry for minimal residual disease (MRD) were performed on January 13, 2026. No obvious abnormal cells were identified by bone marrow microscopy. MRD analysis included the enumeration of 47,875 nucleated bone marrow cells, with only ten abnormal phenotypic mast cells detected. According to IWG-MRT/ECNM unified response criteria for advanced SM, the patient achieved clinical improvement with partial hematologic recovery (8). The patient’s vital signs remained stable, organ function recovered gradually, and they were discharged from the hospital on January 14, 2026. Regular outpatient follow-ups were conducted for four months post-discharge, and the patient maintained a stable general condition without any signs of disease progression.

## 3 Discussion

MCL is a rare, highly aggressive subtype of SM. Its diverse clinical manifestations primarily arise from the abnormal proliferation and tissue infiltration of mast cells, coupled with the release of numerous inflammatory mediators, such as histamine, heparin, and tryptase. Typical clinical features of MCL include fever, night sweats, fatigue, unexplained weight loss, sudden and severe flushing, diarrhea, or anaphylactic shock (9). Meanwhile, extensive infiltration of malignant cells into organs, such as the bone marrow, liver, and spleen, can lead to significant hepatosplenomegaly, severe cytopenia, and bone destruction (10). As reported in several studies and evidenced in our patient, clinical presentations can also include back pain, bleeding, hemolysis, or disseminated intravascular coagulation (11).

The immunophenotype of mast cells in typical MCL includes consistent expression of tryptase and *KIT* (CD117), with aberrant expression of CD25, CD2, or CD30, which serve as hallmarks of neoplastic mast cells, of which CD25 is the most consistently expressed marker (12). However, the immunophenotype of the malignant mast cells in our case was atypical, characterized by CD25 negativity and a lack of tryptase expression by immunohistochemistry. Recent studies have indicated that CD25 negativity is enriched in patients who lack KIT kinase domain mutations, particularly those with juxtamembrane variants (13), consistent with our KIT p.V559G−positive case. The absence of tryptase on routine IHC does not necessarily indicate complete loss of synthesis. Krauth et al. (14) reported that low−level expression may be detected with high−sensitivity IHC. Thus, the tryptase negativity in our patient likely reflects sub−threshold expression rather than absolute deficiency. These atypical immunophenotypic features overlap with several other hematologic entities, including aggressive SM, well-differentiated SM, reactive mastocytosis, acute basophilic leukemia, myelomastocytic leukemia, and acute myeloid leukemia. Therefore, accurate distinction from these disorders is critical (**Table 2**).

**Table 2:** Key diagnostic and differential diagnostic points of the present case and related diseases.

Disease entity	Key immunophenotype	Infiltration pattern	Cytological features	*KIT* mutation
Present case	CD117^bri^, CD203c^+^, CD2^+^, CD25^-^, CD34^-^, HLA-DR^-^, Tryptase^-^	Diffuse infiltration	Atypical mast cells (bilobed, multilobed)	p.V559G
Classic Mast Cell Leukemia	CD117^bri^, CD203c^+^, CD2^+/-^, CD25^+^ (commonly), CD34^-^, Tryptase^+^	Diffuse infiltration	Immature, atypical mast cells	D816V
Aggressive Systemic Mastocytosis	CD117^bri^, CD203c^+^, CD2^+/-^, CD25^+^ (commonly), CD34^-^, Tryptase^+^	Multifocal, dense infiltration	Immature, atypical mast cells	D816V
Well-Differentiated Systemic Mastocytosis	CD117^bri^, CD2^-^, CD25^-^, Tryptase^+^	Multifocal, dense infiltration	Mature, spindle-shaped mast cells	Germline near-membrane domain mutations (e.g., K509I, A533D)
Reactive Mastocytosis	CD117^bri^, CD2^-^, CD25^-^, Tryptase^+^	Scattered, interstitial infiltration	Mature mast cells	None
Acute Basophilic Leukemia	CD123^+^, CD203c^+^, CD117^dim/-^, CD25^-^, Tryptase^+/-^	Diffuse, homogeneous infiltration	Blasts ≥20% and basophils ≥40%	None
Myelomastocytic Leukemia	CD117^+^, CD25^-^, CD2^-^, MPO^-^, Tryptase^+^ (atypical mast cell component)	Diffuse, interstitial infiltration	Metachromatic blasts >10%	None
Acute Myeloid Leukemia	CD34^+^, HLA-DR^+^, CD117^+/-^, CD2^-^, CD25^-^, CD203c^-^, Tryptase^+/-^	Diffuse, homogeneous infiltration	Blasts ≥20%	None

Tryptase positivity or negativity was determined by immunohistochemistry.MPO, myeloperoxidase.

Alternative (non-D816V) *KIT* mutations are more frequently observed in MCL than in the general SM population (15). However, the *KIT* p.V559G (formerly V560G) mutation is exceedingly rare, with only a single case report documented in mast cell sarcoma (16). Recent case reports have described other rare juxtamembrane domain KIT mutations, such as p.V560D in SM-related diseases (17). Similar to p.V559G, these mutations cause ligand-independent activation of the KIT receptor and are molecularly distinct from the hotspot p.D816V mutation. The *KIT* p.V559G mutation is located in the juxtamembrane domain and leads to ligand-independent dimerization and activation of the CD117 receptor. In contrast to the common *KIT* D816V mutation, which confers resistance to imatinib, juxtamembrane domain mutations are typically sensitive to this agent (18). The rationale for imatinib therapy in patients with non-canonical, juxtamembrane KIT mutations is further supported by Alvarez-Twose et al. (19), who demonstrated the efficacy of imatinib in systemic mastocytosis patients lacking exon 17 KIT mutations. Nevertheless, within the context of MCL, the overall efficacy of even an imatinib-sensitive mutation remains uncertain due to the highly aggressive nature of the disease.

Although prior single-center studies enriched for advanced SM reported cytogenetic abnormalities in 15–20% of SM patients (20), the large unselected European Competence Network on Mastocytosis registry cohort demonstrated that karyotypic aberrations are overall rare in real-world SM, even in aggressive subtypes (21). Complex karyotypes, particularly those involving abnormalities such as 5/5q-, 7/7q-, +8, +9, and +19, are well-established adverse prognostic factors (5). The karyotype in the present case was remarkably complex, involving aneuploidy of at least eight chromosomes. A high degree of genomic instability strongly predicts chemotherapy resistance and rapid disease progression. In addition, the presence of monosomy 7 and a complex karyotype raises the possibility of SM associated with myeloid neoplasm, as defined by the ICC 2022 classification (7). Although no definitive morphologic or molecular evidence of concomitant myeloid neoplasm was identified at diagnosis, the possibility of subsequent secondary myeloid clonal diseases cannot be excluded and warrants vigilant long-term monitoring.

MCL treatment remains a formidable clinical challenge. While midostaurin and avapritinib are standard for *KIT* D816V mutations, imatinib was the rational choice for our patient with a *KIT* p.V559G variant. However, the patient’s advanced age, poor performance status, and high-risk complex karyotype rendered any systemic therapy extremely high-risk with unpredictable efficacy. Complex karyotypes are often associated with additional molecular events that can lead to rapidly acquired resistance to targeted therapies. A comprehensive approach was used to manage the patient. Along with imatinib-targeted therapy, concurrent intensive management involving anti-infective, hemostatic, anti-inflammatory, and supportive measures was provided for severe complications, including severe pneumonia, sepsis, gastrointestinal bleeding, and acute myocardial infarction. Thus, although a complete formal response assessment per IWG−MRT/ECNM criteria was limited by the absence of serial serum tryptase measurements and a short follow−up duration, the best categorization of response within this framework is clinical improvement with partial hematological recovery (8). The patient remained stable during the 4−month post−discharge follow−up, an outcome that already exceeded initial expectations. However, given the limited follow−up time, long−term survival benefit cannot be confirmed. In view of the persistent high−risk features, continuous monitoring of bone marrow MRD and organ function is essential for early detection of relapse.

This case highlights that for older patients with MCL with high-risk features and rare *KIT* mutations, treatment must be highly individualized. A tailored therapeutic strategy should be employed to avoid aggressive regimens without clear benefits. Furthermore, the careful management of complications and comprehensive supportive care throughout the treatment course are indispensable for enhancing treatment tolerance and potentially extending survival.

## Data Availability

The original contributions presented in the study are included in the article/supplementary material. Further inquiries can be directed to the corresponding authors.
